# High-Resolution Imaging for the Analysis and Reconstruction of 3D Microenvironments for Regenerative Medicine: An Application-Focused Review

**DOI:** 10.3390/bioengineering8110182

**Published:** 2021-11-10

**Authors:** Michail E. Klontzas, Alexandros Protonotarios

**Affiliations:** 1 Department of Medical Imaging, University Hospital of Heraklion, 71110, Heraklion, Crete, Greece; 2 Computational Biomedicine Laboratory, Institute of Computer Science, Foundation for Research and Technology (FORTH), 70013 Heraklion, Crete, Greece; 3 Department of Radiology, School of Medicine, Voutes Campus, University of Crete, 71003 Heraklion, Crete, Greece; 4 Institute of Cardiovascular Science, University College London, London WC1E 6BT, UK; alexandros.protonotarios.10@ucl.ac.uk

**Keywords:** tissue engineering, regenerative medicine, cardiovascular, musculoskeletal, neural, MRI, microscopy, CT, optical coherence tomography, PET

## Abstract

The rapid evolution of regenerative medicine and its associated scientific fields, such as tissue engineering, has provided great promise for multiple applications where replacement and regeneration of damaged or lost tissue is required. In order to evaluate and optimise the tissue engineering techniques, visualisation of the material of interest is crucial. This includes monitoring of the cellular behaviour, extracellular matrix composition, scaffold structure, and other crucial elements of biomaterials. Non-invasive visualisation of artificial tissues is important at all stages of development and clinical translation. A variety of preclinical and clinical imaging methods—including confocal multiphoton microscopy, optical coherence tomography, magnetic resonance imaging (MRI), and computed tomography (CT)—have been used for the evaluation of artificial tissues. This review attempts to present the imaging methods available to assess the composition and quality of 3D microenvironments, as well as their integration with human tissues once implanted in the human body. The review provides tissue-specific application examples to demonstrate the applicability of such methods on cardiovascular, musculoskeletal, and neural tissue engineering.

## 1. Introduction

The rapid evolution of regenerative medicine and its associated scientific fields, such as tissue engineering, has provided great promise for multiple applications where replacement and regeneration of damaged or lost tissue is needed [1,2,3]. In order to evaluate and optimise the tissue engineering techniques, visualisation of the material of interest is crucial. This includes monitoring of the cellular behaviour, extracellular matrix composition, scaffold structure, and other crucial elements of biomaterials [4]. 

Monitoring of 3D microenvironments can be approached in multiple ways that include microelectromechanical systems and implantable biosensors, which can provide valuable information, but can be hindered by the issues arising from the interaction between the tissue and the foreign body/sensor [5]. Non-invasive visualisation techniques can offer an alternative way to monitor the cell microenvironment in various study models in vitro, ex vivo, or even in vivo. Furthermore, imaging techniques are the way forward for the evaluation of biomaterials in patients as part of a regenerative medicine treatment [6].

In this manuscript, we review both laboratory- and clinical-grade high-resolution imaging techniques and their applications, for the analysis and reconstruction of 3D microenvironments in regenerative medicine.

## 2. Overview of High-Resolution Imaging Techniques

### 2.1. Laboratory-Grade Methods

#### 2.1.1. Contrast Microscopy

Phase-contrast microscopy (PCM) is an optical/bright-field microscopy technique that converts phase shifts in light passing through an imaging subject to brightness changes in the image [7]. The phase difference of light is transformed into an amplitude difference detectable by the human eye, which proves to be a great improvement in contrast compared to regular bright-field microscopy. However, PCM requires relatively transparent imaging subjects; thus, its utility for 3D tissues is limited. Staining methods are available; however, they are often toxic to the cells [7]. 

More recently, it has become feasible to map the absolute phase of light through a variation of PCM, known as quantitative phase imaging (QPI) [8]. High-resolution 3D tomography of live cells has been achieved with QPI via the utilisation of light interferometry in order to achieve depthwise optical gating and reduced noise [9]. In addition to morphological characterisation, QPI may also quantify thermal fluctuations of cell membranes, which can provide functional information in the analysis of cell cultures [10]. 

#### 2.1.2. Confocal Microscopy

Confocal microscopy (CM) is one of the most well-known and widely used techniques for high-resolution imaging in tissues [7]. CM can provide improved sectioning at different depth levels as compared to PCM, and this allows for 3D reconstruction of the tissue. This is achieved by a spatial diameter-controlled pinhole that can limit diffraction. CM can be utilised without any labelling (confocal reflectance microscopy), which is very useful for living cells and tissues. This has been primarily used for the study of extracellular structures [11].

**Figure 1:**
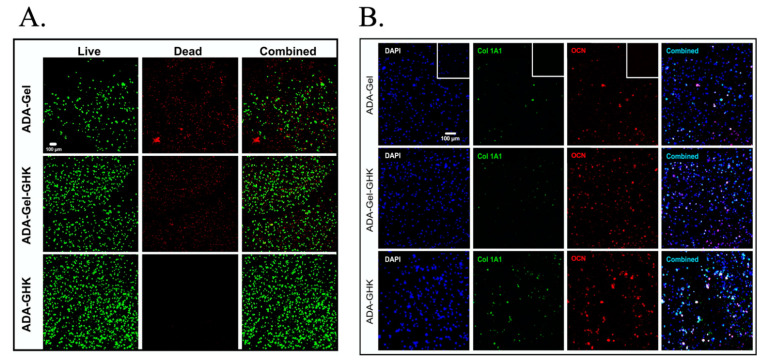
Confocal microscopy images demonstrating umbilical cord blood stem cells encapsulated in different hydrogels with oxidised alginate (ADA), with and without gelatine (Gel) or the GHK peptide (GHK). Confocal microscopy was able to assess the viability in different groups with the use of calcein AM (**A**), as well as the expression of proteins related to osteogenic differentiation (**B**), such as collagen type I (Col1A1) and osteocalcin (OCN). Images were adapted/reprinted from *Acta Biomaterialia*, 88: 224–240, Klontzas ME et al., Oxidized alginate hydrogels with the GHK peptide enhance cord blood mesenchymal stem cell osteogenesis: A paradigm for metabolomics-based evaluation of biomaterial design (2019) [16], https://doi.org/10.1016/j.actbio.2019.02.017, with permission from Elsevier.

CM can prove to be a very powerful source of information for tissues when paired with fluorescent labelling (confocal florescence microscopy). Optical filters allow for the selective excitation and detection of specific wavelengths according to the labelled components chosen by the researcher; this allows for overlaid visualisation of multiple structures, which makes it valuable for 3D structures [12]. Fluorescent CM has found multiple applications within tissue engineering, including collagen formation [13], characterisation of hybrid polymer scaffolds [14], and even angiogenesis imaging [15] (Figure 1).

#### 2.1.3. Multiphoton Microscopy

In contrast to CM, where a single photon excites a fluorescent label in a sample, in multiphoton microscopy (MPM, two or more photons are absorbed by the label at the same instance. This allows the use of lower wavelength photons that are able to penetrate deeply with minimal photodamage, allowing for higher depth-resolution imaging [17]. 

The most common form of MPM in use is two-photon fluorescence microscopy (2-PFM), which utilises near-infrared light with a wavelength twice as long as that required for excitation of the fluorescent molecule [18]. 2-PFM is able to leverage the autofluorescence of certain cellular structures without the need for fluorescent labelling [19]; this has found multiple uses in tissue engineering, ranging from cell viability studies [20] to imaging of the extracellular matrix and elastic fibres of tissue-engineered heart valves [21].

Second-harmonic generation microscopy (SHGM) also relies on two-photon illumination, but the imaging contrast arises from the induced popularisation instead of the excitation emission mechanism [22]. This method is significantly more energy efficient compared to CM and 2-PFM and, thus, leads to less photo-bleaching and heating effects. SHGM has also been used to characterise collagen organisation and spatial organisation of collagen fibres [23,24]. Combinations of 2-PFM and SHGM have also been used in the study of collagen hydrogels and bone formation [25,26].

#### 2.1.4. Optical Coherence Tomography

Optical coherence tomography (OCT) is a high-resolution cross-sectional imaging technique that is based on interferometry [27]. The infrared laser source of OCT allows for significant depth penetration up to several millimetres in real time, and has been known as a type of “optical biopsy” [27]. Structural and functional iterations of OCT exist.

Structural OCT offers the ability to differentiate 3D microstructure and morphology during engineered tissue development, as well as to detect cell dynamics such as migration, proliferation, detachment, and interactions with materials [28]. Structural OCT has also shown utility in skin repair analysis by distinguishing between different layers of skin equivalents, which can be practically applied when evaluating skin equivalents prior to transplantation [29]. OCT can be also applied endoscopically for intravascular, gastrointestinal, or respiratory tract assessment, as it allows for the visualisation of the cellular lining [30,31].

Optical coherence elastography (OCE) utilises OCT to analyse tissue biomechanics by providing measures of tissue elasticity [32]. The imaging contrast is obtained from the mechanical response of the tissue to stimulus, as assessed by mechanical parameters. This allows the study of cell proliferation by analysing the differences in elasticity [33]. This could also prove useful in assessing the state of the failing myocardium as the elasticity parameters are altered in failing hearts [34].

Integration of the Doppler frequency shift to calculate the velocity information for functional blood flow imaging is the aim of Doppler OCT (DOCT). This can provide information-rich 3D flow imaging information with high spatial and temporal resolution. In tissue engineering, this can be useful in the study of microflow in porous scaffolds [35]. Finally, speckle variance OCT (SVOCT) is able to demonstrate 3D mapping of the vascular network inside the tissue by relying on the endogenous blood perfusion to form the contrast [36]. This can be of particular value for the assessment of engineered blood vessels, but also to image the neo-vascularisation as induced by proangiogenic factors [37].

#### 2.1.5. Photoacoustic Microscopy

Photoacoustic microscopy (PAM) is a hybrid in vivo imaging technique that acoustically detects optical contrast via the photoacoustic effect [38]. Both the excitation light and the ultrasonic detection are focused on the sample of interest, and this leads to 3D imaging based on the mechanical scanning of the excitation and detection beams [38]. PAM offers a scalable solution with a high optical absorption contrast that may characterise tissue-engineered samples. Acoustic resolution PAM has been used to quantify the number of melanoma cells and their proliferation profile [39]. Acoustic resolution PAM has also been used to image vascular networks within scaffolds [40]. In contrast, optical resolution PAM can achieve a much higher lateral resolution within the submicron level; this has shown improved imaging of vasculature networks within scaffolds [41]. 

#### 2.1.6. Raman Spectroscopy

Raman spectroscopy (RS) is a non-invasive, high-resolution imaging technique that allows one to obtain biochemical and structural information from biological materials [42]. RS exploits the phenomenon of inelastic scattering (Raman effect). RS can be used to characterise cellular processes such as cell cycle dynamics and cell differentiation [43]. This has been used with foetal osteoblasts, but also with live human alveolar epithelial type II cells in culture [44]. RS has also been used to characterise biomaterial surfaces [45], such as in biocompatibility studies of different implant coatings and their integration with bones [46], or to monitor extracellular matrix formation in three-dimensional scaffolds [47]. 

### 2.2. Clinical-Grade Methods

The use of clinical-grade imaging methods for the visualisation of artificial tissues is imperative for the clinical translation of tissue engineering applications. Clinical-grade imaging methods include computed tomography (CT), magnetic resonance imaging (MRI), and positron emission tomography (PET), as well as hybrid methods such as PET–MRI and PET–CT [6,48]. Such methods are widely used in everyday clinical practice, and can be utilised at several stages of artificial tissue development, starting from the design of the tissue construct, and continuing with the in vitro monitoring of the pre-implantation maturation period and the in vivo monitoring of post-implantation integration with surrounding tissues [49]. Utilisation of these methods in the development of products for regenerative medicine has the potential to accelerate FDA approval by demonstrating post-implantation safety and efficacy, while providing direct correlation between the lab-designed construct properties and the long-term properties of the implanted tissues compared to the surrounding normal human tissue environment. 

#### 2.2.1. Computed Tomography (CT)

**Figure 2:**
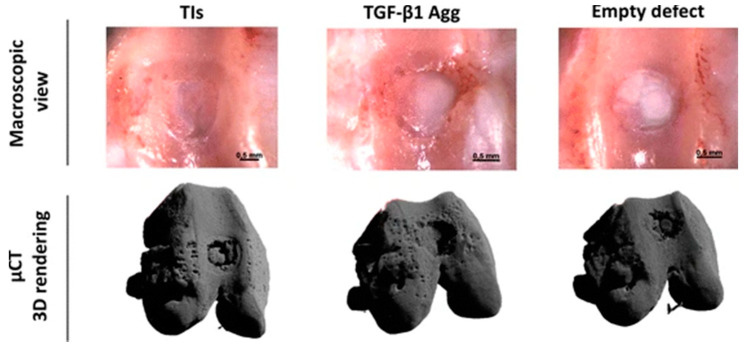
Figure demonstrating how μCT can be used to monitor healing of critical-sized osteochondral defects filled with tissue intermediates (TIs) or transforming growth factor (TGF)-β1 aggregates (Agg) of human periosteum-derived progenitor cells. Adapted under a Creative Commons CC BY license from Mendes LF et al. Stem Cell Res. Ther., 2018; 9 (42) https://doi.org/10.1186/s13287-018-0787-3 [51].

Clinical-grade computed tomography is an X-ray-based method allowing for a spatial resolution of approximately 0.5–0.625 mm with contemporary scanners. In the context of tissue engineering, CT has been used in the form of micro CT (μCT) for the analysis of a multitude of materials and lab-created tissues, with a great emphasis on bone. CT is inferior to other techniques in visualising soft tissues; however, it provides superb resolution for the study of mineralised tissues. The rapid acquisition speed of state-of-the-art scanners enables the collection of time-dependent data before and after the administration of contrast medium, which enables the visualisation of vascularised tissues and the in-depth analysis of the vascular tree. An important advantage of CT is its lower cost and wider availability compared to other clinical-grade techniques, which can enable faster translation of CT-based applications [50] (Figure 2).

#### 2.2.2. Magnetic Resonance Imaging (MRI)

MRI is the imaging modality of choice for the depiction of soft tissues. The inherent superb soft tissue contrast in combination with the absent radiation burden has rendered MRI the key imaging modality for a great variety of applications, including neurological and musculoskeletal disease [52,53]. The magnetic field strength of most clinical-grade magnets ranges between 1.5 T and 3 T, with 7 T systems being slowly introduced in hospitals. This is an important difference from research-grade magnets, which routinely utilise higher magnetic field strengths of up to 11.7 T. Such systems provide excellent resolution and contrast in laboratory samples, but are not suitable for clinical use due to the augmentation of motion artifacts and the exertion of a series of physiological effects on patients, including dizziness, increased temperature, and cardiac conduction disturbance [53,54]. 

Advanced molecular MRI applications, such as diffusion and perfusion MRI and magnetisation transfer, enable a more comprehensive study of tissue structure. Diffusion-weighted imaging (DWI) enables the quantification of water diffusivity at three dimensions, which varies depending on the type of tissue examined. Apparent diffusion coefficient (ADC) maps provide quantitative information (*b*-values) on water molecule diffusion and spatial anisotropy [55]. 

#### 2.2.3. PET and Hybrid Imaging

Positron emission tomography (PET)—as a standalone method, or in hybrid versions such as PET–CT and PET–MRI—represents the ultimate state-of-the-art imaging method that can provide molecular information on the metabolic activity of tissues and the activity of molecular pathways. PET can be used to detect radiolabelled stem cells with 18F-FDG, and cell adhesion peptides such as RGD have been labelled in order to demonstrate cell adhesion and angiogenesis in tissue engineering applications [56,57]. However, the resolution of PET is limited at the macroscale level, and cannot yet be reliably used for the microscale analysis required for the development of artificial tissues. Current use of PET and hybrid applications is limited to the macroscale study of the tissues post-implantation in vivo, which is beyond the scope of this review.

**Figure 3:**
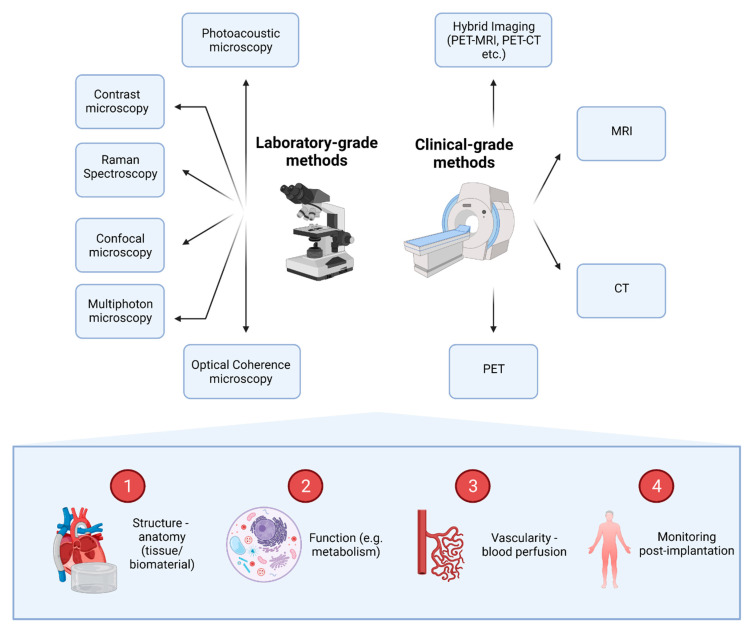
Imaging methods available for the evaluation of 3D tissues and the information that they can provide. A series of laboratory-grade and clinical-grade methods can be utilised to provide information on four main domains. Images can demonstrate anatomical and functional information (e.g., metabolic information). In addition, imaging can reveal and quantify tissue vascularity and perfusion of blood, while providing information about the fate of constructs post-implantation, uncovering potential complications.

An overview of the most commonly used imaging methods and the information that they can provide for 3D tissue microenvironments can be found in Figure 3.

## 3. Tissue-Specific Applications

### 3.1. Cardiovascular Tissue Engineering

#### 3.1.1. Engineered Heart Tissue Imaging

Engineered heart tissue (EHT) approaches have proliferated over the past decade as an emerging technology of disease modelling and drug screening [58]. Synthetic cardiac structures have allowed research beyond the channelopathy paradigm with human pluripotent stem cells, as cardiomyocytes achieve a higher maturity status in three-dimensional configurations. Features such as contractility, force, and other biophysical parameters can be studied. Optical microscopy methods are used to study the morphology and function of EHTs. In heart-on-a-chip (HoC) devices, an optical window can be incorporated to study voltage- or calcium-sensitive dyes that allow for the study of cardiomyocyte physiology [59]. Properties such as contraction can be assessed with relative pixel displacement or as absolute force of contraction [60]. Traction force microscopy can also be used in monolayered cell cultures [59].

Engineered heart muscle (EHM) has been increasingly used in models for cardiac regenerative therapies. EHM transplantation has been shown to lead to long-term cell survival, maintaining high engraftment rates and reduced disease progression [61,62]. Accurate assessment of the transplanted EHMs may be achieved via cardiac strain analysis, using tagged magnetic resonance imaging to assess functional changes in rat models following localised regenerative therapies [63]; this is likely not feasible with conventional measures of global systolic performance.

#### 3.1.2. In Vivo Microscopy of the Heart

Intravital microscopy (IVM) has been shown to provide real-time imaging of cellular processes, and can achieve deep tissue imaging at a high resolution. Although cardiac IVM can be challenging to implement due to difficulties in reaching and immobilising the heart [64], it can provide better quality data than in vitro or ex vivo techniques that cannot mimic the native physiological environment [65]. Cardiac IVM allows the study of both cardiomyocyte metabolism and electrophysiological properties under physiological and pathological conditions, such as ischaemia–reperfusion and myocardial infarction [65,66]. Furthermore, localised events such as white blood cell trafficking have been previously studied [67]. 

### 3.2. Musculoskeletal Tissue Engineering 

Evaluation of engineered musculoskeletal tissues is primarily performed by means of MRI and CT, due to the aforementioned properties of MRI that enable excellent soft tissue contrast (including the depiction of cartilage), and the ability of CT to visualise mineralised bone tissue. 

#### 3.2.1. Bone Tissue Engineering

In terms of bone tissue engineering, a wide range of preclinical studies have utilised MRI to monitor the bone development and maturation in a variety of materials, including metals (e.g., titanium alloys), ceramics (e.g., TiO_2_), and polymers (e.g., collagen, PEG, etc.) [68]. Preclinical studies have employed labelling of stem cells with supramagnetic iron oxide nanoparticles, which are used to localise the cells and confirm their incorporation with the produced extracellular matrix. Such nanoparticles appear with low signal intensity in T2-w sequences inside the labelled stem cells, which regain their normal signal in case of cell death or migration [69,70,71]. When hydroxyapatite is deposited and bone maturation occurs, it appears with low signal intensity in all pulse sequences. The appearance of cancellous bone on MRI greatly depends on the cellular and acellular content between bone trabeculae. MRI signal depends on the balance between the water and fat contents of the construct, the number of labelled cells present, the type of scaffold used, the number of dead cells, and the presence of local inflammation [6]. Advanced confocal microscopy techniques can enable the in vitro cell-by-cell characterisation of the bone stroma in artificial tissues [72]; however, clinical-grade methods do not afford such a level of detail. Novel ultrashort echo time sequences (echo time 100–1000 times shorter than regular sequences) in high-field preclinical and clinical-grade MRI systems allow for the evaluation of the bone itself, which is not feasible with conventional MRI [73].

CT can provide information on the structure of cortical and cancellous mineralised bone at a sub-millimetre level of detail in clinical-grade systems. CT has the ability to demonstrate complications such as subtle fracture lines on implanted tissues and the development of soft tissue inside or outside the contrast. However, due to the low soft tissue contrast, its clinical use is limited to the evaluation of lesions, which primarily affect the dense mineralised bone, and in terms of artificial tissues is mainly limited to fractures and the assessment of the mineralisation patterns of the tissue [74,75]. CT can also provide information on tissue vascularisation with the use of perfusion techniques, which can also be reliably assessed with the use of MRI or intravital microscopy [75,76]. This represents a major shortcoming of preclinical studies, which largely utilize μCT to assess tissue growth. The information provided by such μCT studies has limited application in the clinical setting, and does not promote the direct translation of the findings to humans, which requires the utilisation of purely clinical-grade methods [6]. 

#### 3.2.2. Cartilage Tissue Engineering

With regards to the evaluation of cartilage, MRI is the modality of choice for the assessment of cartilage matrix. MRI has found several applications in preclinical and clinical settings for the evaluation of cartilage regeneration and cartilage damage, as well as the assessment of complications related to surgical procedures aiming at cartilage repair. Imaging protocols used for the assessment of cartilage include a variety of common sequences, including proton density (PD) weighted with spectral fat saturation, T1-w, T2-w, and gradient echo sequences (GRE). In PD-w sequences, cartilage appears with lower signal intensity in comparison with the surrounding joint fluid [77]. In addition, MR arthrography (MRA) can be utilised to assess changes in the cartilage matrix and cartilage damage post-implantation. MRA protocols can be direct (injection of paramagnetic contrast inside a joint) or indirect (intravenous injection and joint loading), with direct MRA protocols being the most commonly used in clinical practice [78]. Novel MRI sequences can quantify the content of cartilage in glycosaminoglycans and proteoglycans, providing a sensitive measure of cartilage degeneration. Such sequences include delayed gadolinium-enhanced MRI of cartilage (dGEMRIC) and T1ρ sequences, the former of which allows the quantification of gadolinium uptake from cartilage glycosaminoglycans, and the latter the quantification of the decrease in proteoglycans seen over time in cartilage degeneration. T2 maps can also allow for the evaluation of cartilage hydration and collagen content [79,80]. Therefore, MRI can provide a multitude of data at the molecular level which can indicate cartilage development and damage in the setting of artificial tissues. All of the aforementioned techniques can be directly translated from the preclinical setting to use in humans, since they have been widely used in clinical practice. In clinical practice, CT arthrography can also provide information on cartilage damage, which is particularly useful for claustrophobic patients or patients with contraindications for MRI (e.g., incompatible metal implants). However, it must be taken into account that CT arthrography can present lesions only on the surface of cartilage, and not deep inside the cartilage matrix, as it relies on the difference in attenuation between the intra-articularly injected contrast and the cartilage [81]. 

### 3.3. Neural Tissue Engineering

Regenerative approaches for nervous system injuries address crucial challenges, particularly since both the central and peripheral nervous systems have limited capacity for self-regeneration. Neural tissue engineering has sought to provide biocompatible structures that can be integrated into the surrounding tissue and can lead to recovery of functionality [82]. Both natural and synthetic biomaterials have been used to create scaffolds; visualisation of those scaffolds and the generated nerve tissue remains challenging.

Electron microscopy techniques such as scanning electron microscopy (SEM) are widely used for the characterisation of scaffolds in nerve tissue engineering, as they can reveal morphological features, pore size, fibre diameter, and other visual estimates of interconnectivity [50]. SEM can be combined with focused ion beam lithography to achieve high-resolution cross-sections of neurites and neurons [83]. Transmission electron microscopy (TEM) can achieve nanometre-scale visualization of nerve tissues and allow for the reconstruction of small, highly detailed sample volumes by tilting ultrathin slices [50].

Confocal laser microscopy (CLSM) techniques have been used to obtain high-resolution optical images at selected depths, and have also been applied in in vivo studies, such as in corneal nerve regeneration [84]. With the use of fluorescent bioactive molecules, drug delivery monitoring can also be achieved [50]. Secretion of extracellular matrix proteins can also be evaluated with the use of CLSM [16]. 

μCT is an alternative, non-destructive method to study the microstructures of objects [85]. In neural tissue engineering, this can be very useful for in vitro or in vivo assessment of the scaffolds on repeated timepoints. This is, however, limited due to the low-density properties of the neural tissue, leading to limited distinguishing capability. X-ray staining techniques may be useful, as there are dyes available that can be applied to stain soft tissues or low-density scaffold polymers, such as osmium tetroxide, lyophilic salts, and gold [50,51]. μCT techniques remain complementary to the classic approaches.

**Figure 4:**
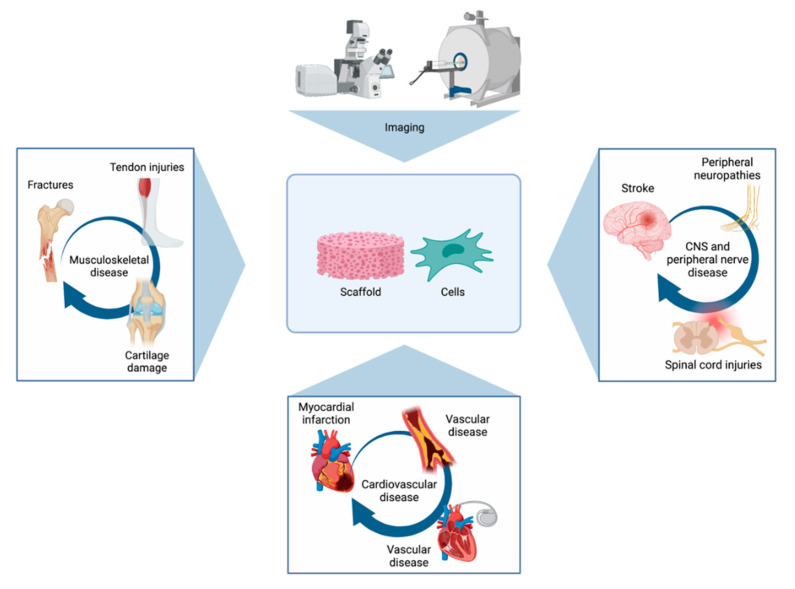
Figure presenting some of the most important tissue-specific applications of imaging methods. Imaging can be used to visualise the repair of a series of musculoskeletal, cardiovascular, and neural conditions with the use of scaffolds and/or stem cells.

An overview of important tissue-specific applications of imaging methods can be found in Figure 4.

## 4. Conclusions

Imaging plays a vital role in the development and clinical use of artificial tissues. Tissue engineers have a wide arsenal of imaging methods that can assess tissues at various scales. Structural and functional information can be provided to demonstrate the quality and functionality of tissues meant to be used for clinical purposes, which is of vital importance for regulatory body approval and commercial licensing. Imaging can also be used to detect potential complications post-implantation. The majority of the imaging methods presented in this review can be used for a variety of applications—alone, or in combination with other imaging methods. The selection of method(s) to be used greatly depends on the scale that we wish to visualise, with laboratory-grade methods being more commonly used at the micro level, whereas clinical-grade methods are most commonly used in vivo to visualise structures at a millimetre or centimetre scale. For all of these reasons, the familiarity of laboratory scientists and clinicians with all available imaging modalities, as well as collaboration between engineers, radiologists, and other clinicians, is of utmost importance in order to facilitate mutual understanding and rapid clinical translation.

## Data Availability

Not applicable.

## References

[1] Pigeot S., Klein T., Gullotta F., Dupard S.J., Garcia-Garcia A., García-García A., Prithiviraj S., Lorenzo P., Filippi M., Jaquiery C. (2021). Manufacturing of human tissues as off-the-shelf grafts programmed to induce regeneration. Adv. Mater..

[2] López-Martínez S., Rodríguez-Eguren A., de Miguel-Gómez L., Francés-Herrero E., Faus E., Díaz A., Ferrero H., Cervelló I., Pellicer A. (2021). Bioengineered endometrial hydrogels with growth factors promote tissue regeneration and restore fertility in murine models. Acta Biomater..

[3] Lin W., Kluzek M., Iuster N., Shimoni E., Kampf N., Klein J. (2020). Cartilage-inspired, lipid-based boundary-lubricated hydrogels. Science.

[4] Wang S., Larina I.V., Narayan R.J. (2017). High-Resolution Imaging Techniques in Tissue Engineering.

[5] Barthes J., Ozcelik H., Hindie M., Ndreu-Halil A., Hasan A., Vrana N.E. (2014). Cell microenvironment engineering and monitoring for tissue engineering and regenerative medicine: The recent advances. BioMed Res. Int..

[6] Klontzas M.E., Kakkos G.A., Papadakis G.Z., Marias K., Karantanas A.H. (2021). Advanced clinical imaging for the evaluation of stem cell based therapies. Exp. Opin. Biol. Ther..

[7] Smith L.E., Smallwood R., Macneil S. (2010). A comparison of imaging methodologies for 3D tissue engineering. Microsc. Res. Tech..

[8] Popescu G., Park Y.K., Choi W., Dasari R.R., Feld M.S., Badizadegan K. (2008). Imaging red blood cell dynamics by quantitative phase microscopy. Blood Cells Mol. Dis..

[9] Kim T., Zhou R., Mir M., Sevket Derin M., Scott Carney P., Goddard L.L., Popescu G. (2014). White-light diffraction tomography of unlabelled live cells. Nat. Photonics.

[10] Park Y.K., Best C.A., Badizadegan K., Dasari R.R., Feld M.S., Kuriabova T., Kuriabova M.L., Levine A.J., Popescu G. (2010). Measurement of red blood cell mechanics during morphological changes. Proc. Natl. Acad. Sci. USA.

[11] Gillette B.M., Rossen N.S., Das N., Leong D., Wang M., Dugar A., Sia S.K. (2011). Engineering extracellular matrix structure in 3D multiphase tissues. Biomaterials.

[12] Pampaloni F., Reynaud E.G., Stelzer E.H.K. (2007). The third dimension bridges the gap between cell culture and live tissue. Nat. Rev. Mol. Cell Biol..

[13] Krahn K.N., Bouten C.V.C., Van Tuijl S., Van Zandvoort M.A.M.J., Merkx M. (2006). Fluorescently labeled collagen binding proteins allow specific visualization of collagen in tissues and live cell culture. Anal. Biochem..

[14] Sahoo S., Cho-Hong J.G., Siew-Lok T. (2007). Development of hybrid polymer scaffolds for potential applications in ligament and tendon tissue engineering. Biomed. Mater..

[15] Moon J.J., Saik J.E., Poché R.A., Leslie-Barbick J.E., Lee M.-H., Smith A.A., Dickinson M.E., West J.L. (2010). Biomimetic hydrogels with pro-angiogenic properties. Biomaterials.

[16] Klontzas M.E., Reakasame S., Silva R., Morais J.C.F., Vernardis S., MacFarlane R.J., Heliotis H.M., Tsiridis E., Panoskaltsis N., Boccaccini A.R. (2019). Oxidized alginate hydrogels with the GHK peptide enhance cord blood mesenchymal stem cell osteogenesis: A paradigm for metabolomics-based evaluation of biomaterial design. Acta Biomater..

[17] Larson A.M. (2011). Multiphoton microscopy. Nat. Photonics.

[18] Palikaras K., Tavernarakis N. (2012). Multiphoton Fluorescence Light Microscopy.

[19] Monici M. (2005). Cell and tissue autofluorescence research and diagnostic applications. Biotechnol. Ann. Rev..

[20] Dittmar R., Potier E., Van Zandvoort M., Ito K. (2012). Assessment of cell viability in three-dimensional scaffolds using cellular auto-fluorescence. Tissue Eng. Part C.

[21] Konig K., Schenke-Layland K., Riemann I., Stock U.A. (2005). Multiphoton autofluorescence imaging of intratissue elastic fiber. Biomaterials.

[22] Campagnola P. (2011). Second harmonic generation imaging microscopy: Applications to diseases diagnostics. Anal. Chem..

[23] Akilbekova D., Bratlie K.M. (2015). Quantitative characterization of collagen in the fibrotic capsule surrounding implanted polymeric microparticles through second harmonic generation imaging. PLoS ONE.

[24] Kabir M.M., Inavalli V.V.G.K., Lau T.-Y., Toussaint K.C. (2013). Application of quantitative second-harmonic generation microscopy to dynamic conditions. Biomed. Opt. Exp..

[25] Raub C.B., Suresh V., Krasieva T., Lyubovitsky J., Mih J.D., Putnam A.J., Tromberg B.J., George S.C. (2007). Noninvasive assessment of collagen gel microstructure and mechanics using multiphoton microscopy. Biophys. J..

[26] Villa M.M., Wang L., Huang J., Rowe D.W., Wei M. (2013). Visualizing osteogenesis in vivo within a cell-scaffold construct for bone tissue engineering using two-photon microscopy. Tissue Eng. Part C.

[27] Fujimoto J.G., Pitris C., Boppart S.A., Brezinski M.E. (2000). Optical coherence tomography: An emerging technology for biomedical imaging and optical biopsy. Neoplasia.

[28] Liang X., Graf B.W., Boppart S.A. (2009). Imaging engineered tissues using structural and functional optical coherence tomography. J. Biophotonics.

[29] Spoler F., Forst M., Marquardt Y., Hoeller D., Kurz H., Merk H., Abuzahra F. (2006). High-resolution optical coherence tomography as a non-destructive monitoring tool for the engineering of skin equivalents. Skin Res. Technol..

[30] Yabushita H., Bouma B.E., Houser S.L., Thomas Aretz H., Jang I.-K., Schlendorf K.H., Kauffman C.R., Shishkov M., Kang D.H., Halpern E.F. (2002). Characterization of human atherosclerosis by optical coherence tomography. Circulation.

[31] Tearney G.J., Brezinski M.E., Bouma B.E., Boppart S.A., Pitris C., Southern J.F., Fujimoto J.G. (1997). In vivo endoscopic optical biopsy with optical coherence tomography. Science.

[32] Kennedy B.F., Kennedy K.M., Sampson D.D. (2014). A review of optical coherence elastography: Fundamentals, techniques and prospects. IEEE J. Sel. Top. Quantum Electron..

[33] Ko H.-J., Tan W., Stack R., Boppart S.A. (2006). Optical coherence elastography of engineered and developing tissue. Tissue Eng..

[34] Wang S., Singh M., Tran T.T., Leach J., Aglyamov S.R., Larina I.V., Martin J.F., Larin K.V. (2018). Biomechanical assessment of myocardial infarction using optical coherence elastography. Biomed. Opt. Exp..

[35] Veksler B., Kobzev E., Bonesi M., Meglinski I. (2008). Application of optical coherence tomography for imaging of scaffold structure and micro-flows characterization. Laser Phys. Lett..

[36] Mariampillai A., Standish B.A., Moriyama E.H., Khurana M., Munce N.R., Leung M.K.K., Jiang J., Cable A., Wilson B.C., Vitkin A. (2008). Speckle variance detection of microvasculature using swept-source optical coherence tomography. Opt. Lett..

[37] Hsu C.W., Poché R.A., Saik J.E., Ali S., Wang S., Yosef N., Calderon G.A., Larry S., Vadakkan T.G., Larina I.V. (2015). Improved angiogenesis in response to localized delivery of macrophage-recruiting molecules. PLoS ONE.

[38] Yao J., Wang L.V. (2012). Photoacoustic microscopy. Laser Photonics Rev..

[39] Zhang Y., Cai X., Choi S.W., Kim C., Wang L.V., Xia Y. (2010). Chronic label-free volumetric photoacoustic microscopy of melanoma cells in three-dimensional porous scaffolds. Biomaterials.

[40] Cai X., Zhang Y., Li L., Choi S.-W., Matthew R.M., Yao J., Kim C., Xia Y., Wang L.V. (2013). Investigation of neovascularization in three-dimensional porous scaffolds in vivo by a combination of multiscale photoacoustic microscopy and optical coherence tomography. Tissue Eng. Part C.

[41] Hu S., Wang L.V. (2013). Optical-resolution photoacoustic microscopy: Auscultation of biological systems at the cellular level. Biophys. J..

[42] Butler H.J., Ashton L., Bird B., Cinque G., Curtis K., Dorney J., Esmonde-White K., Fullwood N.J., Gardner B., Martin-Hirsch P.L. (2016). Using Raman spectroscopy to characterize biological materials. Nat. Prot..

[43] Swain R.J., Jell G., Stevens M.M. (2008). Non-invasive analysis of cell cycle dynamics in single living cells with Raman micro-spectroscopy. J. Cell. Biochem..

[44] Jell G., Notingher I., Tsigkou O., Notingher P., Polak J.M., Hench L.L., Stevens M.M. (2008). Bioactive glass-induced osteoblast differentiation: A noninvasive spectroscopic study. J. Biomed. Mater. Res. Part A.

[45] Perlaki C.M., Liu Q., Lim M. (2014). Raman spectroscopy based techniques in tissue engineering-an overview. Appl. Spectrosc. Rev..

[46] Bertoluzza A., Fagnano C., Tinti A., Morelli M.A., Tosi M.R., Maggi G., Marchetti P.G. (1994). Raman and infrared spectroscopic study of the molecular characterization of the biocompatibility of prosthetic biomaterials. J. Raman Spectrosc..

[47] Kunstar A., Leferink A.M., Okagbare P.I., Morris M.D., Roessler B.J., Otto C., Karperien M., van Blitterswijk C.A., Moroni L., van Apeldoorn A.A. (2013). Label-free Raman monitoring of extracellular matrix formation in three-dimensional polymeric scaffolds. J. R. Soc. Interface.

[48] Klontzas M.E., Papadakis G.Z., Marias K., Karantanas A.H. (2020). Musculoskeletal trauma imaging in the era of novel molecular methods and artificial intelligence. Injury.

[49] Klontzas M.E., Karantanas A.H. (2019). MR imaging of artificial musculoskeletal tissues: Bridging the gap between basic science and clinical reality. Hell. J. Radiol..

[50] Zhu N., Chen X., Chapman D. (2010). A brief review of visualization techniques for nerve tissue engineering applications. J. Biomim. Biomater. Tissue Eng..

[51] Mendes L.F., Katagiri H., Tam W.L., Chai Y.C., Geris L., Roberts S.J., Luyten F.P. (2018). Advancing osteochondral tissue engineering: Bone morphogenetic protein, transforming growth factor, and fibroblast growth factor signaling drive ordered differentiation of periosteal cells resulting in stable cartilage and bone formation in vivo. Stem Cell Res. Ther..

[52] Karantanas A.H. (2014). What’s new in the use of MRI in the orthopaedic trauma patient?. Injury.

[53] Rutland J.W., Delman B.N., Gill C.M., Zhu C., Shrivastava R.K., Balchandani P. (2020). Emerging use of ultra-high-field 7T MRI in the study of intracranial vascularity: State of the field and future directions. AJNR Am. J. Neuroradiol..

[54] Ladd M.E., Bachert P., Meyerspeer M., Moser E., Nagel A.M., Norris D.G., Schmitter S., Speck O., Straub S., Zaiss M. (2018). Pros and cons of ultra-high-field MRI/MRS for human application. Prog. Nucl. Magn. Reson. Spectrosc..

[55] Schaefer P.W., Grant P.E., Gonzalez R.G. (2000). Diffusion-weighted MR imaging of the brain. Radiology.

[56] Tamura M., Unno K., Yonezawa S., Hattori K., Nakashima E., Tsukada H., Nakajima M., Oku N. (2004). In vivo trafficking of endothelial progenitor cells their possible involvement in the tumor neovascularization. Life Sci..

[57] Collignon A.M., Lesieur J., Anizan N., Azzouna R.B., Poliard A., Gorin C., Letourneur D., Chaussain C., Rouzet F., Rochefort G.Y. (2018). Early angiogenesis detected by PET imaging with 64Cu-NODAGA-RGD is predictive of bone critical defect repair. Acta Biomater..

[58] Tzatzalos E., Abilez O.J., Shukla P., Wu J.C. (2016). Engineered heart tissues and induced pluripotent stem cells: Macro- and microstructures for disease modeling, drug screening, and translational studies. Adv. Drug Deliv. Rev..

[59] Stein J.M., Mummery C.L., Bellin M. (2021). Engineered models of the human heart: Directions and challenges. Stem Cell Rep..

[60] Van Meer B.J., Tertoolen L.G.J., Mummery C.L. (2016). Concise review: Measuring physiological responses of human pluripotent stem cell derived cardiomyocytes to drugs and disease. Stem Cells.

[61] Zimmermann W.H., Melnychenko I., Wasmeier G., Didié M., Naito H., Nixdorff U., Hess A., Budinsky L., Brune K., Michaelis B. (2006). Engineered heart tissue grafts improve systolic and diastolic function in infarcted rat hearts. Nat. Med..

[62] Riegler J., Tiburcy M., Ebert A., Tzatzalos E., Raaz U., Abilez O.J., Shen Q., Kooreman N.G., Neofytou E., Chen V.C. (2015). Human engineered heart muscles engraft and survive long term in a rodent myocardial infarction model. Circ. Res..

[63] Qin X., Riegler J., Tiburcy M., Zhao X., Chour T., Ndoye B., Nguyen M., Adams J., Ameen M., Denney T.S. (2016). Magnetic resonance imaging of cardiac strain pattern following transplantation of human tissue engineered heart muscles. Circ. Cardiovasc. Imaging.

[64] Vaghela R., Arkudas A., Horch R.E., Hessenauer M. (2021). Actually seeing what is going on—Intravital microscopy in tissue engineering. Front. Eng. Biotechnol..

[65] Vinegoni C., Aguirre A.D., Lee S., Weissleder R. (2015). Imaging the beating heart in the mouse using intravital microscopy techniques. Nat. Prot..

[66] Aguirre A.D., Vinegoni C., Sebas M., Weissleder R. (2014). Intravital imaging of cardiac function at the single-cell level. Proc. Natl. Acad. Sci. USA.

[67] Ueno T., Kim P., McGrath M.M., Yeung M.Y., Shimizu T., Jung K., Sayegh H., Chandraker A.K., Abdi R., Yun S.H. (2016). Live images of donor dendritic cells trafficking Via CX3CR1 pathway. Front. Immunol..

[68] De Witte T.-M., Fratila-Apachitei L.E., Zadpoor A.A., Peppas N.A. (2018). Bone tissue engineering via growth factor delivery: From scaffolds to complex matrices. Regen. Biomater..

[69] Lalande C., Miraux S., Derkaoui S.M., Mornet S., Bareille R., Fricain J.C., Franconi J.M., Visage C.L., Letourneur D., Amédée J. (2011). Magnetic resonance imaging tracking of human adipose derived stromal cells within three-dimensional scaffolds for bone tissue engineering. Eur. Cells Mater..

[70] Klontzas M.E., Karantanas A.H. (2020). Considerations on the use of ferumoxytol-enhanced MRI for tracking stem cell implants in cartilage defects. Radiology.

[71] Theruvath A.J., Nejadnik H., Lenkov O., Yerneni K., Li K., Kuntz L., Wolterman C., Tuebel J., Burgkart R., Liang T. (2019). Tracking stem cell implants in cartilage defects of minipigs by using ferumoxytol-enhanced MRI. Radiology.

[72] Allenby M.C., Okutsu N., Brailey K., Guasch J., Zhang Q., Panoskaltsis N., Mantalaris A. (2021). A spatiotemporal microenvironment model to improve design of a 3D bioreactor for red cell production. Tissue Eng. Part A.

[73] Ma Y.J., Jerban S., Jang H., Chang D., Chang E.Y., Du J. (2020). Quantitative Ultrashort Echo Time (UTE) magnetic resonance imaging of bone: An update. Front. Endocrinol..

[74] Lin E., Alessio A. (2009). What are the basic concepts of temporal, contrast, and spatial resolution in cardiac CT?. J. Cardiovasc. Comput. Tomogr..

[75] Subhawong T.K., Fishman E.K., Swart J.E., Carrino J.A., Attar S., Fayad M. (2010). Soft-tissue masses and masslike conditions: What does CT add to diagnosis and management?. AJR Am. J. Roentgenol..

[76] Gaustad J.V., Brurberg K.G., Simonsen T.G., Mollatt C.S., Rofstad E.K. (2008). Tumor vascularity assessed by magnetic resonance imaging and intravital microscopy imaging. Neoplasia.

[77] Naraghi A., White L.M. (2015). MRI of labral and chondral lesions of the hip. AJR Am. J. Roentgenol..

[78] Schmid M.R., Nötzli H.P., Zanetti M., Wyss T.F., Hodler J. (2003). Cartilage lesions in the hip: Diagnostic effectiveness of MR arthrography. Radiology.

[79] Jungmann P.M., Baum T., Bauer J.S., Karampinos D.C., Erdle B., Link T.B., Li X., Trattnig S., Rummeny E.J., Woertler K. (2014). Cartilage repair surgery: Outcome evaluation by using noninvasive cartilage biomarkers based on quantitative MRI techniques?. BioMed Res. Int..

[80] Kondo S., Nakagawa Y., Mizuno M., Katagiri K., Tsuji K., Kiuchi S., Ono H., Muneta T., Koga H., Sekiya I. (2019). Transplantation of aggregates of autologous synovial mesenchymal stem cells for treatment of cartilage defects in the femoral condyle and the femoral groove in microminipigs. Am. J. Sport Med..

[81] Perdikakis E., Karachalios T., Katonis P., Karantanas A. (2011). Comparison of MR-arthrography and MDCT-arthrography for detection of labral and articular cartilage hip pathology. Skelet. Radiol..

[82] Doblado L.R., Martínez-Ramos C., Pradas M.M. (2021). Biomaterials for neural tissue engineering. Front. Nanotechnol..

[83] Greve F., Frerker S., Bittermann A.G., Burkhardt C., Hierlemann A., Hall H. (2007). Molecular design and characterization of the neuron-microelectrode array interface. Biomaterials.

[84] Lagali N., Griffith M., Fagerholm P., Merrett K., Huynh M., Munger R. (2008). Innervation of tissue-engineered recombinant human collagen-based corneal substitutes: A comparative in vivo confocal microscopy study. Investig. Ophthalmol. Vis. Sci..

[85] Bonse U., Busch F. (1996). X-ray computed microtomography (μCT) using synchrotron radiation (SR). Prog. Biophys. Mol. Biol..

